# A Bioinspired Functionalization of Polypropylene Separator for Lithium-Sulfur Battery

**DOI:** 10.3390/polym11040728

**Published:** 2019-04-22

**Authors:** Zhijia Zhang, Xuequan Li, Yawen Yan, Wenyi Zhu, Li-Hua Shao, Junsheng Li

**Affiliations:** 1 School of Chemistry, Chemical Engineering and Life Sciences, Wuhan University of Technology, Wuhan 430070, China; ZJ_Zhang1112@163.com (Z.Z.); superjuniorslh@163.com (Y.Y.); 15207172760@163.com (W.Z.); 2 Institute of Solid Mechanics, Beihang University, Beijing 100083, China; lixuequan@buaa.edu.cn

**Keywords:** separator, lithium-sulfur battery, functionalization, shuttle effect

## Abstract

Lithium-sulfur batteries have received intensive attention, due to their high specific capacity, but the shuttle effect of soluble polysulfide results in a decrease in capacity. In response to this issue, we develop a novel tannic acid and Au nanoparticle functionalized separator. The tannic acid and gold nanoparticles were modified onto commercial polypropylene separator through a two-step solution process. Due to a large number of phenolic hydroxyl groups contained in the modified layer and the strong polarity of the gold nanoparticles, the soluble polysulfide generated during battery cycling is well stabilized on the cathode side, slowing down the capacity fade brought by the shuttle effect. In addition, the modification effectively improves the electrolyte affinity of the separator. As a result of these benefits, the novel separator exhibits improved battery performance compared to the pristine polypropylene separator.

## 1. Introduction

The ever-increasing demand for a secondary battery with high energy density has significantly promoted the development of Lithium-Sulfur (Li-S) battery. Through electrode and/or electrolyte engineering, Li-S battery can deliver a much higher discharge capacity in the initial charge/discharge cycles compared to that for a typical lithium ion battery [1,2,3]. However, the cyclability of a Li-S battery has yet to be improved to meet the performance requirements posed by practical applications, such as electric vehicles. The root for the poor cyclability of a Li-S battery is the shuttle of soluble polysulfides (LiPSs) between the electrodes, which not only causes a rapid loss of active S, but also accelerates the failure of the battery [4]. Thus, the key to further enhance the performance of a Li-S battery is to prevent the shuttling of LiPSs [5].

Functionalization of the separator with a barrier layer against soluble LiPSs is a cost-effective approach toward high-performance Li-S battery [6,7,8,9,10,11]. The functionalized barrier layer could act like a sieve that prevents the transport of soluble LiPSs through the membrane physically [12,13]. When using a conductive matrix for such functionalization, the functionalized barrier layer could further enhance the battery performance by acting as a secondary current collector [14,15]. Despite the effectiveness of such functionalization, it is also recognized that only physical blocking of soluble LiPSs might not be sufficient to secure a complete suppression of shuttling. The chemically active component is suggested to incorporate into the functionalization layer to further restrict the shuttling of soluble LiPSs [16]. These active components provide strong anchoring sites for stabilization of soluble LiPSs [17,18]. In addition, they also propel the conversion of soluble LiPSs, which further improves battery performance [19,20,21]. Therefore, a composite functionalization layer that offers both physical and chemical interactions with soluble LiPSs is favorable for separator modification of a Li-S battery [22,23,24,25,26].

Tannic acid (TA) is a widely-existing natural polyphenol with both high surface affinity and redox ability [27,28,29]. The high surface affinity of TA can be used for the modification of PP separator to endow the separator with physical barrier properties. The redox property of TA can be exploited to generate sulphiphilic nanoparticles on the TA coating to further block soluble LiPSs. Inspired by these merits of TA, we develop a bioinspired functionalization of polypropylene (PP) separator, which results in a modified separator with a tannic acid/Au functionalization layer. The composite separator effectively suppresses LiPSs shuttling and enhances the electrolyte affinity of the PP substrate, thus improving the performance.

## 2. Materials and Methods

To fabricate the functionalized separator, commercial PP separator (Celgard 2400, thickness: 25 μm; pore size: 0.043 μm; porosity: 41%) was pretreated by immersing in methanol solution for 30 min, followed by washing and subsequent incubation in Tris-HCl (FEIYANG BIO, Xi’an, China) buffer solution (pH 8.5) of tannic acid (5 mg·mL^−1^) (Alfa Aesar, Lancashire, England). The solution was stirred gently at room temperature for 24 h to form a uniform coating layer. The residual tannic acid was washed away with deionized water. Finally, the separator was dried in a vacuum oven at 40 °C for 24 h to obtain a tannic acid-modified PP separator (abbreviated as PP-TA in the following). In the second step, the PP-TA separator was directly immersed in an aqueous solution of HAuCl_4_ (0.2 mg·mL^−1^) (Innochem, Beijing, China) and stirred slowly for 24 h. Finally, the modified separator was rinsed with deionized water and dried at 40 °C for 24 h. The mass of the coating layer, determined by weighting, was ~0.10 mg·cm^−2^.

The surface characteristic functional groups of the modified separator were confirmed by Fourier transformed infrared spectroscopy (FT-IR, Nicolet AVATAR 370) with a resolution of 4 cm^−1^. The TGA measurement was conducted with Thermogravimetric analysis equipment (SDT Q600). The measurement was conducted from room temperature to 800 °C in the air, at a ramping rate of 10 °C per minute. The wettability of the separators was tested by a contact angle apparatus. A water droplet of 1 μL was used for each measurement. The surface of the separator was subjected to X-ray a Bruker D8 Advance diffractometer (D/MAX-RB RU-200B, Rigaku) with a Cu Κα radiation (λ=1.5406Å) to determine the crystal structure of the modified layer (scan rate: 10° min^−1^). The surface morphology of the modified separator was characterized by electron microscopy (SEM) in SE2 mode with an accelerating voltage of 10 kV.

The performance of lithium-sulfur battery was characterized by CR-2016 type cells. To prepare the sulfur cathode, pure sulfur, Super P, and LA133 binder were mixed in a ratio of 6:3:1, and an appropriate amount of deionized water was added as a solvent. Then, the mixed slurry was pasted on the aluminum foil and dried at 50 °C for 24 h. Finally, the cathode was cut into 10 mm electrode pieces. The loading of the active material of the electrode sheet was controlled to be 1.0–1.5 mg·cm^−2^. The electrolyte used for the cell assembling was 1 M LiTFSI in 1:1 (*v*/*v*) 1,2-dimethoxyethane (DME) and 1,3-dioxacyclopentane (DOL) with 0.1 M LiNO_3_ as additive. The electrolyte content of the battery was controlled to be 25 μL·mg^−1^ sulfur. For the ionic conductivity test, stainless steel/electrolyte-soaked separator/stainless steel cell was assembled, and the measurement was conducted on an Autolab (PG 302N) workstation (frequency range: 10^5^–10^−2^ Hz, amplitude: 5 mV). To test the electrochemical stability of the separator, a “lithium foil/separator/stainless steel” cell was first assembled and linear sweep voltammetry (LSV) was then collected using the CHI 660D electrochemical workstation. The test voltage window was 1-6 V and the scan rate was set to be 5 mV s^−1^. The cycle performance of Li-S batteries was characterized by LAND battery test system (Wuhan, China) and the voltage window was 1.5–2.8 V (current density was 0.2 C (1 C = 1,675 mA·g^−1^)). The cyclic voltammetry test (CV) is consistent with the battery cycle performance test conditions with a sweep rate of 0.1 mV/s. Electrochemical impedance spectroscopy (EIS) spectra of the batteries were measured on the Autolab workstation from 10^5^ to 10^−2^ Hz with an amplitude of 5 mV.

## 3. Results

**Figure 1:**
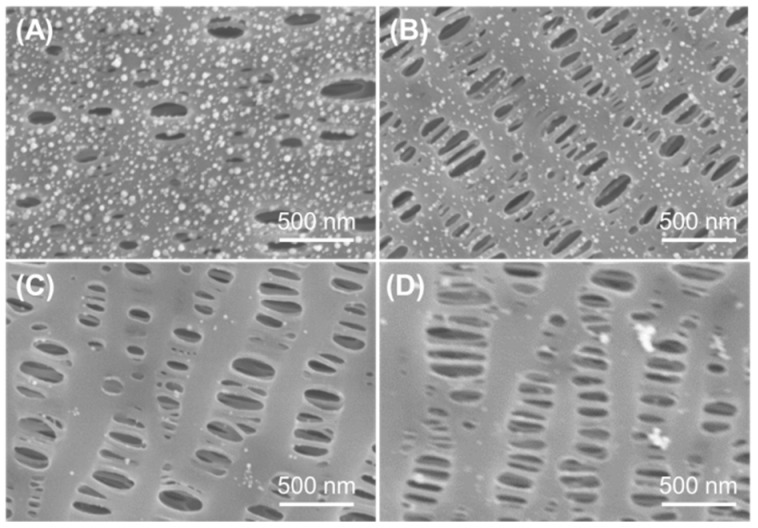
SEM images of PP-TA/Au separator prepared in HAuCl_4_ bath with different pH values: (**A**) pH 4; (**B**) pH 6.8; (**C**) pH 7.6 and (**D**) pH 8.3.

**Figure 2:**
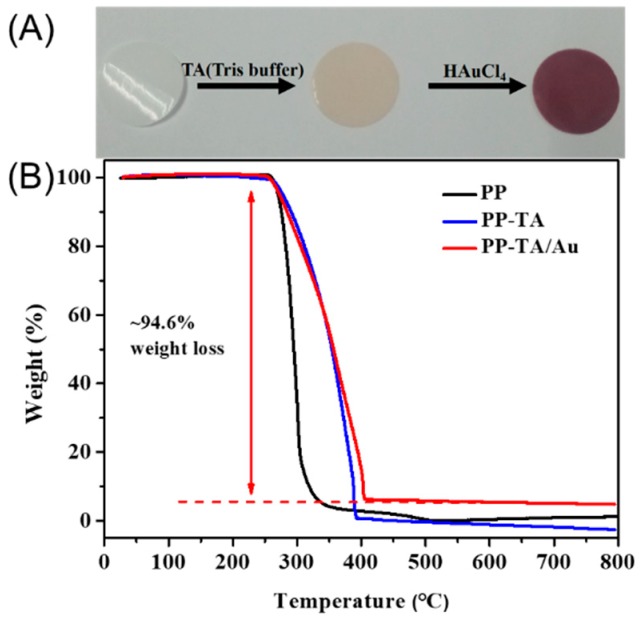
(**A**) picture of pristine polypropylene (PP) separator (left), PP-TA separator (middle) and PP-TA/Au separator (right). (**B**) TG curve of PP separator, PP-TA separator and PP-TA/Au separator.

The tannic acid and Au nanoparticles functionalized PP separator (PP-TA/Au) was prepared with a two-step process. In the first step, TA was modified onto the PP substrate through a simple solution process without any pretreatment of the separator. Normally, binder-free surface functionalization of a PP separator requires a pretreatment process to introduce active sites for subsequent functionalization, due to the inert nature of PP. Benefiting from the high surface affinity brought by the rich phenol groups, TA can be readily introduced onto the PP separator under a mild condition. In the second step, TA functionalized PP was immersed in a HAuCl_4_ bath for 24 h, resulting in a PP-TA/Au separator. Since the pH value of HAuCl_4_ bath influences the formation of Au nanoparticles on the PP substrate, HAuCl_4_ solutions with different pH values were used and compared. As shown in Figure 1, little Au nanoparticles are formed on PP at alkaline conditions. When the pH value was lowered to 4, dense Au nanoparticles with small sizes were generated on PP without blocking of the pore structures of PP. Thus, we expect that a pH of 4 was the optimal pH and this pH value was used for following investigations. The color change of the separator after each modification step is shown in Figure 2A. The mass loading of TA and Au nanoparticles on PP-TA/Au, determined by weighing the separator after each modification step, was calculated to be 0.06 and 0.10 mg cm^−2^, respectively. Thermogravimetry characterizations show that PP separator experienced a rapid weight loss in the air, until its complete decomposition at 500–600 °C (Figure 2B). For PP-TA/Au separator, a residual weight of ~5.4% is retained after heating up to 800 °C, which indicates that the weight percentage of Au nanoparticle in the composite separator is ~5.4%.

**Figure 3:**
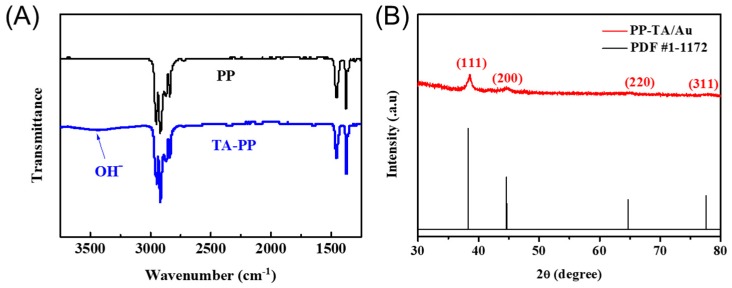
(**A**) FTIR spectra of pristine PP and PP-TA separator. (**B**) XRD spectra of PP-TA/Au separator.

To prove the successful modification of TA on PP in the first step, FTIR measurements were conducted. A broad peak assigned to hydroxyl groups were present on the spectra of TA-PP (Figure 3A), suggesting successful modification of TA onto the PP separator. The presence of Au nanoparticles on the PP-TA/Au was verified with XRD analysis. Characteristic peaks assigned to Au (111), (200), (220) and (311) peak (at 2θ = 38.2°, 44.4°, 64.6° and 77.5°, respectively) were found in the XRD spectra of PP-TA/Au (Figure 3B). [30] The presence of these nanoparticles clearly demonstrates the reductive formation of Au nanoparticles on PP separator. Furthermore, XPS measurements also confirmed the existence of Au on the modified separator. The surface Au concentration determined from XPS was 3.14 at%, close to the result from the TG measurements. 

**Figure 4:**
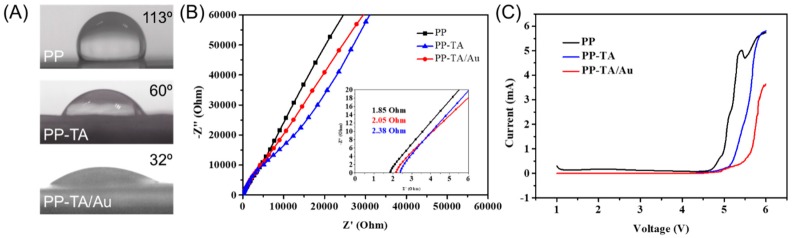
(**A**) Water contact angle of PP, PP-TA and PP-TA/Au separator. (**B**) EIS spectra of stainless steel/electrolyte saturated separator/stainless steel cell assembled with PP, PP-TA and PP-TA/Au separator. (**C**) LSV curve of Li foil/electrolyte saturated separator/stainless steel cell assembled with PP, PP-TA and PP-TA/Au separator.

The effect of functionalization on the separator’s electrolyte affinity was evaluated with a water contact angle (WCA) measurement (Figure 4A). The pristine PP separator had a high WCA of 113°. After TA modification, the separator turned into a hydrophilic one and the WCA was reduced to 60°. When Au nanoparticles are further decorated onto PP-TA, the WCA of the separator lowered down to 32°. The high hydrophilicity of PP-TA/Au suggests that the separator had a high electrolyte affinity, which is important for an improved electrode-electrolyte interface. The ionic conductivity of a separator is crucial for its battery performance. Hence, the conductivity of the separators is characterized by a stainless steel/electrolyte saturated separator/stainless steel cell (Figure 4B). The measured ionic conductivity was 0.76, 0.81 and 0.94 mS·cm^−1^ for PP, PP-TA, PP-TA/Au, respectively. The enhanced ionic conductivity upon TA/Au modification is originated to the improved electrolyte affinity of the functionalization layer. The electrochemical stability of the separator was also assessed by LSV measurements with a Li/electrolyte saturated separator/stainless steel cell (Figure 4C). It was observed that PP-TA and PP-TA/Au separator was stable up to ~5.0 V, securing their application in Li-S battery.

**Figure 5:**
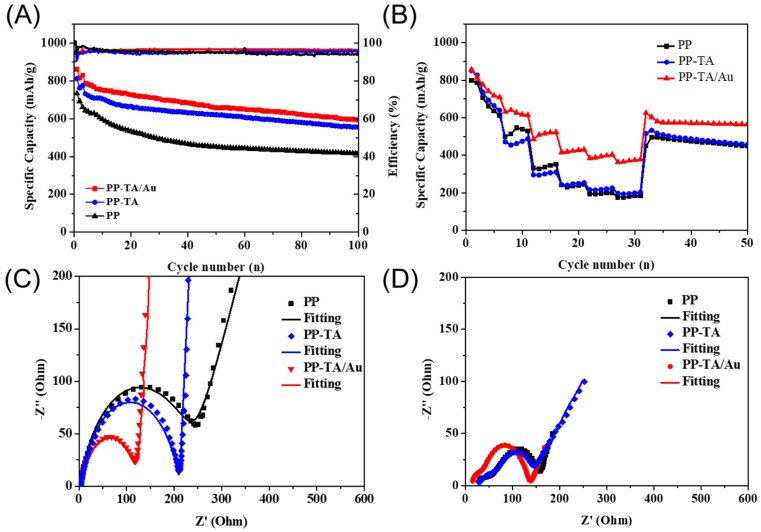
(**A**) Cycling performance of Li-S battery assembled with PP, PP-TA and PP-TA/Au separator. (**B**) Rate performance of Li-S battery assembled with PP, PP-TA and PP-TA/Au separator. (**C**) EIS spectra of uncycled Li-S battery assembled with PP, PP-TA and PP-TA/Au separator. (**D**) EIS spectra of cycled Li-S battery assembled with PP, PP-TA and PP-TA/Au separator.

Li-S battery was assembled with PP separator and functionalized PP separators and characterized to show the benefits of functionalization. The CV curves of the separators were firstly recorded (Figure S1). As shown in the CV curves of PP battery in the first cycle, two cathodic peaks centered at ~2.18 V and 1.71 V were present, which could be ascribed to the reduction of sulfur to Li_2_S_x_ (4 ≤ x ≤ 8) and reduction of to Li_2_S_2_ and Li_2_S.The anodic peak at ~2.55 V corresponded to oxidation of Li_2_S_2_ and Li_2_S to element sulfur. In subsequent cycles, the CV curves tended to overlap with each other, due to the formation of a stable SEI layer on the electrode after the first cycle. Apparently, the battery with PP-TA/Au separator showed better reversibility and the lower gap between anodic peaks and cathodic peaks. These changes indicate that the TA/Au functionalization may improve battery performance. The charge-discharge performance of the separators was tested at 0.2C (Figure 5A). The first cycle discharge capacity of the battery with PP, PP-TA and PP-TA/Au separator was 733.0, 816.9 and 860.8 mAh·g^−1^, respectively. The discharge capacity of the batteries dropped significantly, due to a series of irreversible reactions, such as electrolyte decomposition and SEI formation. During the whole cycling, PP-TA/Au separator shows the highest discharge performance with a capacity of 597.8 mAh·g^−1^ after 100 cycles. The rate performance of the batteries was also quantified (Figure 5B). Battery assembled with pristine PP separator exhibited discharge capacities of 798.6, 513.4, 337.3, 234.9, 197.4, 179.5 mAh·g^−1^ at 0.2 C, 0.5 C, 1 C, 1.5 C and 2 C, respectively. When the battery was cycled back to 0.2 C, a discharge capacity of 495.7 mAh·g^−1^ was retained. PP-TA separator shows similar rate performance with PP separator. In contrast, PP-TA/Au separator had an enhanced discharge performance. Its discharge capacities at 0.2 C, 0.5 C, 1 C, 1.5 C and 2 C were measured to be 850.5, 486.6, 309.8, 246.5, 225.3 and 202.8 mAh·g^−1^, respectively. A high discharge capacity of 533.4 was kept when cycled back to 0.2 C, proving the good reversibility of the battery.

To understand the excellent battery performance of the separator, EIS spectra of the batteries before and after battery cycling was recorded (Figure 5C). The EIS curve of the battery before cycling was composed of a depressed semicircle and an inclined line, which are associated with the charge transfer resistance and diffusion related resistance (Figure 5D). Notably, the battery with PP-TA/Au separator had the lowest charge transfer resistance (117.8 Ω) among the batteries. This result clearly demonstrated that the use of PP-TA/Au separator improved the interface compatibility between the electrode and electrolyte, due to the improved electrolyte affinity of the separator. After cycling, an additional depressed semicircle corresponding to the overall film resistance appeared in the high frequency region of the EIS spectra. The R_film_ and R_ct_ of the battery with PP-TA/Au separator was determined to be 20.29 Ω and 102.1 Ω, respectively, which were lower than those for PP (71.32 Ω and 76.11 Ω). The decreased R_film_ for PP-TA/Au indicates that a more uniform SEM layer was formed in both sulfur cathode and lithium anode, which is possibly resulted from the suppressed shuttling of soluble LiPSs. Such a low R_film_ and small R_ct_ explains the high battery performance of PP-TA/Au.

**Figure 6:**
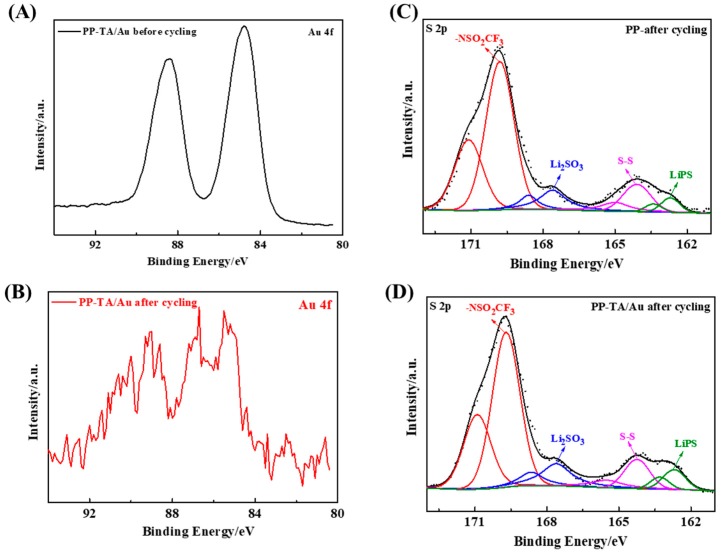
Au 4f spectra of PP-TA/Au separator (**A**) before and (**B**) after cycling in Li-S battery. S 2p spectra of PP-TA/Au separator (**C**) before and (**D**) after cycling in Li-S battery.

Finally, XPS analysis was conducted with the separators disassembled from the cycled battery to gain more insight into the interaction between the functionalization layer and sulfur species. First of all, the peak intensity of the Au 4f peak decrease after battery cycling (Figure 6A,B), suggesting that the Au nanoparticles were still stabilized on the separator after cycling. In addition, the position of Au 4f peak shifted toward high binding energy, which suggests that Au chemically interacts with polysulfide species during battery cycling. Furthermore, the S 2p peak of the cycled PP separator and cycled PP-TA/Au separator was analyzed (Figure 6C,D). As shown in the figure, there are multiple sulfur species of different chemical states identified from the S 2p spectra. A detailed peak fitting proves that PP-TA/Au separator had a higher peak component from lithium polysulfide (LiPS) [7]. This observation directly demonstrates that the functionalization layer helps to anchor the LiPS during battery cycling and explains the improved performance of the functionalized separator. From the results above, it can be concluded that the polysulfide shuttling across PP-TA/Au separator is successfully suppressed. As a result, the battery performance is improved. Compared with the representative functionalization approaches, which includes blade coating, polymerization etc., the current approach relies on a simple solution process, which is advantageous for a cost-effective production process. In addition to its facile functionalization process, the functionalization results in a bifunctional coating that can realize the suppression of shuttling through the synergy of physical blockage and chemical adsorption. Such a strategy is also beneficial for the efficient prevention of polysulfide shuttling.

## 4. Conclusions

A bioinspired functionalization is developed to enhance the electrolyte compatibility, as well as the barrier property of polypropylene separator for Li-S battery application. The novel functionalization is based on the use of a natural polyphenol which has a high surface affinity and reducing capability and can proceed under mild solution condition. Such a functionalization results in a composite separator with a TA/Au layer. Benefiting from the high polarity and excellent LiPSs adsorption capability of the TA/Au layer, Li-S battery assembled with the functionalized separator delivers a much-improved discharge capacity in both cycling tests and rate performance evaluation. Due to the generality, simplicity and the effectiveness of the functionalization method, we expect that the functionalization method reported herein can be potentially used as a general approach for the modification of separators in advanced battery systems.

## Supplementary Materials

The following are available online at https://www.mdpi.com/2073-4360/11/4/728/s1, Figure S1: CV curves of the Li-S battery assembled with PP or PP-TA/Au separator.
