# Supplementary material for: A Bioinspired Functionalization of Polypropylene Separator for Lithium-Sulfur Battery
Source: Polymers (Basel). 2019 Apr 22;11(4):728. doi: 10.3390/polym11040728 (PMC6523575; doi:10.3390/polym11040728)
Supplement: Supplementary file 1 [file polymers-11-00728-s001.pdf]

A bioinspired functionalization of polypropylene separator for lithium sulfur battery

Zhijia Zhang [1], Xuequan Li [2], Yawen Yan [1], Wenyi Zhu [1], Li-Hua Shao [2,*] and Junsheng Li [1,*]

[1] School of Chemistry, Chemical Engineering and Life Sciences, Wuhan University of Technology, Wuhan 430070, P. R. China
[2] Institute of Solid Mechanics, Beihang University, Beijing 100191, PR China

* Correspondence: shaolihua@buaa.edu.cn (L. Sh); li_j@whut.edu.cn (J. Li)

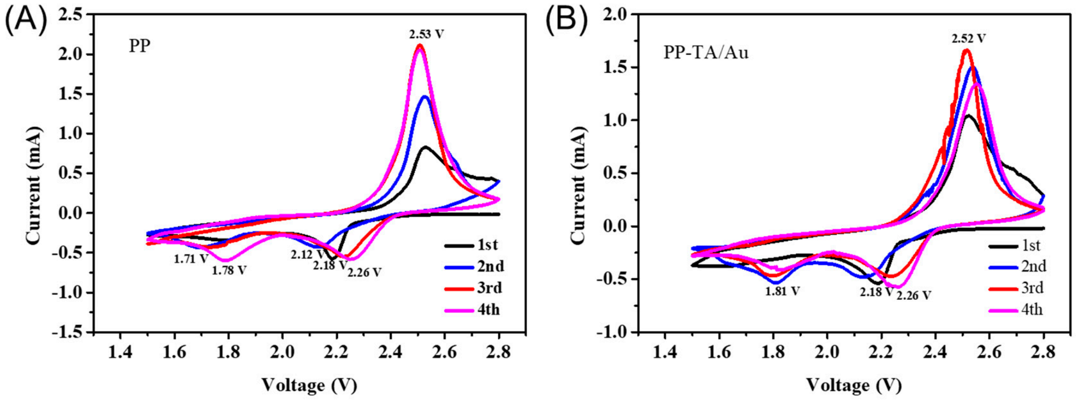

Figure S1. CV curves of the Li-S battery assembled with (A) PP separator or (B) PP-TA/Au separator.
